# The relationship Between Occupation Types, Education, and Volunteer Behaviors Among Older Americans

**DOI:** 10.1093/geroni/igab046.2592

**Published:** 2021-12-17

**Authors:** Patrick Ho Lam Lai, Cal Halvorsen, Christina Matz

**Affiliations:** 1 Boston College School of Social Work, Chestnut Hill, Massachusetts, United States; 2 Boston College, Chestnut Hill, Massachusetts, United States

## Abstract

Research shows that productive engagement in later life, such as paid work and volunteering, is associated with health and wellbeing. From a life-course approach, personal history and experiences developed from occupations earlier in life may affect individual’s willingness and ability to volunteer in later life; reciprocally, volunteering tends to extend networks and roles from their previous work after retirement. Further, education, which influences career development, indirectly affects late-life volunteering. Using data from 329,938 individuals aged 50 to 85 in more than 700 occupations from the Volunteer Supplement of Current Population Survey since 2010, this study found that older adults had higher volunteer rates (40%) when their current or latest jobs require more human interactions, compared to those jobs were mainly characterized as office work (31%) and jobs not requiring much human interaction (21%). More specifically, occupations with higher volunteering rates were more likely to be related to social, educational, or spiritual sectors. Some careers seem to provide skills and networks needed in volunteer roles, such as accounting clerks (ranked 12th in volunteering) and museum technicians (ranked 13th). Diverse educational levels make the relationship between occupation and volunteering more complex. For instance, those older adults without a high school diploma and who have office work as their current or latest job (16%) have higher volunteering rates than those occupations requiring (14%) or not requiring much human interaction (10%). Policymakers can take the human interactions and skills needed in careers and educational levels into account when thinking of strategies to promote volunteering.

